# P-97. Safety, Tolerability, and Immunogenicity of Revaccination With mRNA-1345, an Investigational Vaccine Against RSV, Given at 12 Months Following a Primary Dose in Adults Aged ≥ 50 Years

**DOI:** 10.1093/ofid/ofae631.304

**Published:** 2025-01-29

**Authors:** Jaya Goswami, Jose F Cardona, Denise C Hsu, Alana Simorellis, Lauren Wilson, Rakesh Dhar, Xiaowei Wang, Archana Kapoor, Avi Collins, Vinicius Righi, Lan Lan, Jiejun Du, Honghong Zhou, Sonia K Stoszek, Christine A Shaw, Caroline Reuter, Eleanor Wilson, Jacqueline Miller, Rituparna Das

**Affiliations:** Moderna, Inc., Cambridge, Massachusetts; Indago Research & Health Center, Hialeah, Florida; Moderna, Inc., Cambridge, Massachusetts; Moderna, Inc., Cambridge, Massachusetts; Moderna,. Inc., Cambridge, Massachusetts; Moderna, Inc., Cambridge, Massachusetts; Moderna, Inc., Cambridge, Massachusetts; Moderna, Inc., Cambridge, Massachusetts; Moderna, Inc., Cambridge, Massachusetts; Moderna, Inc., Cambridge, Massachusetts; Moderna, Inc., Cambridge, Massachusetts; Moderna, Inc., Cambridge, Massachusetts; Moderna, Inc., Cambridge, Massachusetts; Moderna, Inc., Cambridge, Massachusetts; Moderna, Inc., Cambridge, Massachusetts; Moderna, Inc., Cambridge, Massachusetts; Moderna, Inc., Cambridge, Massachusetts; Moderna, Inc., Cambridge, Massachusetts; Moderna, Inc., Cambridge, Massachusetts

## Abstract

**Background:**

mRNA-1345 is an investigational RSV vaccine that has demonstrated efficacy for the prevention of lower-respiratory tract disease in older adults. The duration of protection and need for revaccination have not yet been defined. Interim findings are presented from a phase 3 trial evaluating mRNA-1345 revaccination at 12 months after a primary dose in adults aged ≥ 50 years.

Figure 1.

**Figure:**
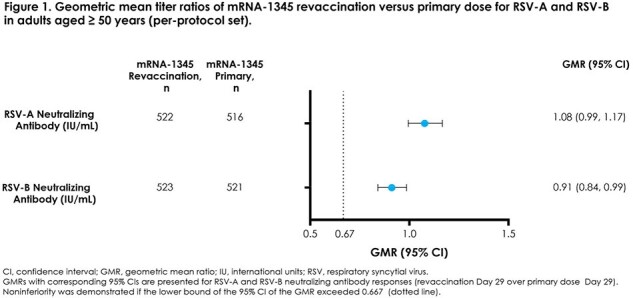

**Methods:**

Part C of a multi-part, ongoing, phase 3 trial (NCT05330975) evaluated the safety, tolerability, and immunogenicity of open-label revaccination with mRNA-1345 (50 µg) in participants aged ≥ 50 years who previously received a primary dose of mRNA-1345 in Part B of the trial. Safety, tolerability, and immunogenicity (RSV-A and RSV-B neutralizing antibody [nAb] responses) of revaccination were primary objectives. Non-inferiority of immune responses was assessed via geometric mean titer ratio (GMR; 95% CI lower bound > 0.667) at Day 29 post-revaccination (Part C) versus Day 29 post-primary dose (Part B). Results of the Day 29 post-revaccination interim analysis are presented here.

**Results:**

Overall, 543 participants were revaccinated with mRNA-1345 (50 µg). Solicited local and systemic adverse reactions within 7 days post revaccination were reported by 55.8% and 50.3% of participants, respectively. Injection site pain (54.3%), headache (33.5%), myalgia (33.5%), fatigue (31.1%), and arthralgia (28.5%) were most frequently reported (≥ 20%) and were primarily grade 1 or 2 in severity. Up to 28 days after revaccination, no adverse events (AEs) led to study discontinuation, and no fatalities or vaccine-related serious AEs, AEs of special interest, or severe AEs were reported. All coprimary immunogenicity endpoints met pre-specified criteria for non-inferiority based on Day 29 GMRs (revaccination Day 29 vs primary Day 29); nAb GMRs (95% CI) were 1.08 (0.99, 1.17) for RSV-A and 0.91 (0.84, 0.99) for RSV-B (**Figure 1**).

**Conclusion:**

Revaccination with mRNA-1345 administered 12 months after a primary dose was well-tolerated, had no identified safety concerns, and elicited RSV nAbs at Day 29 that were non-inferior to those after a primary mRNA-1345 dose in adults aged ≥ 50 years. These data support mRNA-1345 revaccination at 12 months after primary vaccination if the need for revaccination is determined.

**Disclosures:**

**Jaya Goswami, MD**, Moderna, Inc.: Employee|Moderna, Inc.: Stocks/Bonds (Public Company) **Denise C. Hsu, MD**, Moderna, Inc.: Employee|Moderna, Inc.: Stocks/Bonds (Public Company) **Alana Simorellis, PhD**, Moderna, Inc.: Employee|Moderna, Inc.: Stocks/Bonds (Public Company) **Lauren Wilson, MSN**, Moderna, Inc.: Employee|Moderna, Inc.: Stocks/Bonds (Public Company) **Rakesh Dhar, MD**, Moderna, Inc.: Employee|Moderna, Inc.: Stocks/Bonds (Public Company) **Xiaowei Wang, PhD**, Moderna, Inc.: Employee|Moderna, Inc.: Stocks/Bonds (Public Company) **Archana Kapoor, PhD**, Moderna, Inc.: Employee|Moderna, Inc.: Stocks/Bonds (Public Company) **Avi Collins, BScN**, Moderna, Inc.: Employee|Moderna, Inc.: Stocks/Bonds (Public Company) **Vinicius Righi, PharmD, MBA**, Moderna, Inc.: Employee|Moderna, Inc.: Stocks/Bonds (Public Company) **Lan Lan, PhD**, Moderna, Inc.: Employee|Moderna, Inc.: Stocks/Bonds (Public Company) **Jiejun Du, PhD**, Moderna, Inc.: Employee|Moderna, Inc.: Stocks/Bonds (Public Company) **Honghong Zhou, Ph.D.**, Moderna, Inc.: Employee|Moderna, Inc.: Stocks/Bonds (Public Company) **Sonia K. Stoszek, PhD**, Moderna, Inc.: Employee|Moderna, Inc.: Stocks/Bonds (Public Company) **Christine A. Shaw, PhD**, Moderna, Inc.: Employee|Moderna, Inc.: Stocks/Bonds (Public Company) **Caroline Reuter, MD, MSCI**, Moderna, inc.: Employee|Moderna, inc.: Stocks/Bonds (Public Company) **Eleanor Wilson, MD, MHS**, Moderna, Inc.: Employee|Moderna, Inc.: Stocks/Bonds (Public Company) **Jacqueline Miller, MD**, Moderna, Inc.: Employee|Moderna, Inc.: Stocks/Bonds (Public Company) **Rituparna Das, M.D.**, Moderna, Inc.: Employee|Moderna, Inc.: Stocks/Bonds (Public Company)

